# STEPPING DOWN FROM JGH OPEN

**DOI:** 10.1002/jgh3.12851

**Published:** 2022-12-08

**Authors:** Khean‐Lee Goh

**Affiliations:** ^1^ University of Malaya Kuala Lumpur Malaysia

This December issue of the JGH Open marks the swansong of my tenure as Editor in Chief (EiC). We launched the journal in September 2017 [1] and I was the inaugural EiC. The JGH Open is the first “open” journal in the field of gastroenterology in the Asia‐Pacific region and we were somewhat filled with trepidation when we first started. In a blink of an eye, we have now been in publication for 6 years.

One of our greatest concern was the article processing charge (APC) which is levied for all “open” journals. To encourage submissions the Journal of Gastroenterology and Hepatology Foundation (who co‐owns the journal with Wiley) subsidised the APC until 2018. Professor Ian Roberts Thomson, a very close friend, was JGHF chairman and was involved in the discussions in setting up the journal and also took on the role as an associate editor. Ian had also deputize for me in late 2021 and early 2022. His friendship and support throughout my tenure has been invaluable and reassuring.

We started by publishing monthly issues but submissions were few in the beginning and we had to change to bimonthly issues. Slowly, however submissions have increased and in 2021 we reverted back to publishing monthly. The JGH Open is now indexed in the Directory of Open Access Journals (DOAJ), Embase (Elsevier), EBSCO, and PubMed via PMC deposit (NLM) and in the Emerging Sources Citation Index (ESCI) [2]. From 2023 onwards, an impact factor will be given to all journals which are indexed in the ESCI. Our ultimate goal however, is to obtain indexing from Science Citation Index Expanded (SCIE).

I have been ably supported by a team of associate editors, many of whom are friends. Some have left the position over the years but we have had new faces stepping in to replace them. I am truly grateful to each and every one of them for their help and immeasurable contributions to the journal. Professor Vineet Ahuja from the All‐India Institute of Medical Sciences, Delhi takes over the position of EiC from first January 2023. Vineet has been in the editorial board form the beginning. In Vineet, we have a well‐regarded academician who has published widely. He will no doubt bring the journal up to the next level.

The journal has been fortunate in being managed by a very efficient team from Wiley in the day‐to‐day running of the journal. The publication office is in Melbourne with Tamara D'Mello as our Senior Publishing Manager over the past 1 year. The help from Tamara, her predecessors and the publication managers have been tremendous. The editors are truly grateful to be supported by a “professional” team from Wiley.
